# Impact of trimethylaminuria on daily psychosocial functioning

**DOI:** 10.1002/jmd2.12170

**Published:** 2020-10-06

**Authors:** Daniel Roddy, Philomena McCarthy, Darragh Nerney, Jennifer Mulligan‐Rabbitt, Edwin Smith, Eileen P. Treacy

**Affiliations:** ^1^ National Centre for Inherited Metabolic Disorders The Mater Misericordiae University Hospital Dublin Ireland; ^2^ University College Dublin Dublin Ireland; ^3^ Trinity College Dublin The University of Dublin Dublin Ireland; ^4^ Department of Clinical Chemistry Sheffield Children's Hospital Sheffield UK

**Keywords:** trimethylaminuria (TMAU), psychosocial, patients' perspectives, lived experience, coping mechanism, strategic planning

## Abstract

**Background:**

Trimethylaminuria (TMAU) (OMIM #602079) is a rare inherited metabolic condition. TMAU is associated with decreased hepatic trimethylamine N‐oxidation, which leads to an excess of the volatile trimethylamine (TMA) instead of substrate conversion to trimethylamine N‐oxide (TMAO). TMA is a tertiary amine derived from the enterobacterial metabolism of precursors such as choline and phosphatidylcholine present in the diet, and is also a bacterial metabolite of TMAO, a normal constituent of saltwater fish. When the involved enzyme flavin mono‐oxygenase 3 is deficient, TMA builds up and is released in the person's sweat, urine, and breath, giving off a strong body odor. We have recently reported the biochemical and genetic characteristics of 13 Irish adult patients with TMAU attending the main Irish Reference Center. Research on the behavioral and psychosocial aspects of this condition is limited. This study explores the patients' perspectives of living with TMAU in Ireland.

**Methods:**

A qualitative descriptive phenomenological approach was used. Six adults participated in this study. Data were gathered through semi‐structured interviews, which were transcribed and analyzed.

**Results:**

The results suggest that the participants experienced a negative journey to diagnosis. Fear, anxiety, paranoia, and dysfunctional thinking are a constant struggle. Participants reported using avoidant coping mechanisms and strategic planning to navigate daily life.

**Conclusion:**

It is considered that the results from this study will inform future interventions with this unique patient cohort.

**Synopsis**

Trimethylaminuria has a significant impact on the psychosocial functioning of those living with the condition and an emphasis on psychological intervention is recommended.

## 1 BACKGROUND

Trimethylaminuria (TMAU) also known as “fish odor syndrome” (OMIM #602079) is a rare inherited metabolic condition associated with decreased hepatic trimethylamine *N*‐oxidation, which leads to an excess of the volatile trimethylamine (TMA) instead of substrate conversion to TMA *N*‐oxide (TMAO).^1^, ^2^, ^3^ TMA is a tertiary amine derived from the enterobacterial metabolism of precursors such as choline and phosphatidylcholine present in the diet, and is also a bacterial metabolite of TMAO, a normal constituent of saltwater fish. When the involved enzyme flavin mono‐oxygenase 3 (FMO3) is deficient, TMA builds up and is released in the person's sweat, urine, and breath, giving off a strong body odor.^2^, ^3^, ^4^

Loss of function pathogenic variants of the *FMO3* gene, the main FMO liver isoform, present in homozygous or compound heterozygous states are associated with severe primary TMAU. More commonly milder substitutions (polymorphisms) of the *FMO3* gene compounded with environmental factors may cause secondary TMAU. Transient cases are also reported with differing pathogeneses.

We have recently reported the biochemical and genetic characteristics of 13 Irish adult patients with TMAU attending the main Irish Reference Center.^5^ It was concluded that urinary biochemical analysis probably remains the primary diagnostic approach to classify the severity of TMAU, while *FMO3* gene analysis is more informative for the more severe presentations of the disorder.

It is difficult to estimate the true prevalence of TMAU, due to its rarity among the general population. Genetic studies have estimated the incidence of heterozygous carrier for severe TMAU to range from 0.5% to 1% while frequency rates of the severe inherited form are estimated at 1 in 40,000.^2^ In childhood or early adulthood, individuals complain of body odor and/or halitosis and seek care from pediatricians, family doctors, or other medical specialists.^6^

There is no known cure for the condition though treatments are available.^4^ One treatment involves avoiding egg yolks, legumes, red meats, fish, beans, and other foods that contain choline, carnitine, nitrogen, sulfur, and lecithin.^7^ Another treatment is taking short courses of low doses of antibiotics such as neomycin and metronidazole.^8^ Treatment can also include the use of acidic soaps and body lotions, and the daily intake of activated charcoal and copper chlorophyllin.^9^, ^10^

While TMAU is not life threatening, delayed diagnosis, body odor, and the lack of a cure may lead to significant psychosocial issues.^11^, ^12^ Todd highlighted the stigma and psychosocial consequences of TMAU.^13^Affected individuals can experience shame and embarrassment, fail to maintain relationships, avoid contact with people who comment on their condition, and are obsessive about masking the odor with hygiene products or by smoking. The malodourous aspect can have negative effects on education, personal life, career, and relationships.^11^ Social isolation, low self‐esteem, depression, paranoid behavior, and some cases of attempted suicide in people with TMAU have also been reported.^1^, ^14^, ^15^ Rutkowski et al^16^ designed an assessment tool to provide a quantitative measure of treatment efficacy in patients with TMAU, pre‐ and post‐treatment. This examines odor characteristics, social well‐being, health care professionals (HCPs) support, and mental well‐being.

As psychosocial complications of this condition were frequently encountered by the affected patient group, we sought to investigate these issues further as a follow up to our earlier report. The aim of this study was thus to explore psychosocial aspects of adults living with TMAU through semi‐structured interviews. It is hoped this will inform care pathways and help HCPs to formulate effective, person‐centered treatment plans and interventions.

## 2 METHODS

Given the rarity of TMAU and the limited research on psychosocial aspects, a qualitative method with a descriptive phenomenological approach is used.

Urine TMA analysis at initial diagnosis was measured using the method described in Manning et al^17^. A revised TMA analytical method was used for TMA analysis of repeat samples analyzed during the last year. The control reference level for the initial and repeat urine TMA levels are provided in Table 1.

**TABLE 1:** A, Patients' demographic data. B, Patients' genetic and biochemical data

(A)					
ID	Gender and age	Relationship status	Educational status	Mental health	Mental health professional
1	Male 37	Partner	Masters (level 9)	Depression, anxiety	Psychologist, psychiatrist
2	Female 35	Single	Bachelors (level 8)	Depression (not diagnosed)	Counselor (1.5 y)
3	Male 36	Married	Leaving Certificate (level 5)	None	None
4	Male 72	Married	Masters (level 9)	None	None
5	Female 37	Single	Bachelors (level 8)	OCD	Counselor
6	Female 30	Single	Bachelors (level 8)	None	None

(B)			
ID	Initial diagnostic urinary TMA level (at presentation) (μmol/mmol creat)^a^	Most recent TMA level (μmol/mmol creat)^b^	Genotype
1	20.7	0.6	N/A
2	12.1	<2.5	No pathogenic variant detected
3	24.8	40.3	Compound heterozygous for c.458C>T,p.(Pro153Leu) and c.682G>A,p.(Gly228Ser)
4	25.4	0.6	Heterozygous for c.472G>A,p.(Glu158Lys) and c.932A>G,p.(Glu308Gly)
5	11.8	14.4	Compound heterozygous for c.458C>T,p.(Pro153Leu) and c.694G>T,p.(Asp232Tyr)
6	35.2	30.1	Compound heterozygous for c.458C>T,p.(Pro153Leu) and c.989G>A,p.(Gly330Glu)

*Note:* Patients 3 and 6 have intermittent persistent high urinary TMA levels despite a history of adherence to treatment and reported clinical improvement.

Abbreviation: TMA, trimethylamine.

^a^ TMA reference level for initial samples taken at diagnosis (before 2017): range 2.5 to 10.8 μmol/mmol creatinine.

^b^ Updated TMA reference level: range <7.7 μmol/mmol creatinine.

### 2.1 Participants

Twenty patients with a confirmed diagnosis of TMAU attending the National Centre for Inherited Metabolic Disorders–Adult Service (NCIMD) in the Mater Misericordiae University Hospital (MMUH), Ireland, were invited to participate. Ethical approval was granted by the MMUH Ethics Committee. Inclusion criteria included being aged 18 and over, having a confirmed diagnosis of TMAU confirmed by biochemical testing or *FMO3* gene testing, and attending the NCIMD. Exclusion criteria included lacking the capacity to consent.

All the patients in the study were following a choline and TMA restricted diet, and were taking riboflavin 100 mg once daily. Patients were provided with an information sheet, and consent form. Seven patients initially agreed to participate in the study. It was subsequently determined that one patient had transient TMAuria; therefore, six patients continued with the study. Of the remaining six participants, all were Irish Caucasian, three were male and three were female, five were employed, and one retired. Median age for the cohort was 36.5 years (range, 30–75 years). Table 1A,B summarizes the participants' characteristics.

### 2.2 Measures

A demographic questionnaire and semi‐structured interview guide were designed and administered to participants. The interview guide was based on themes that emerged during standard of care support groups for individuals with TMAU. The questions were based around areas of daily living, impact on social, educational, professional, and personal functions, and experiences of HCPs and the health care system.

### 2.3 Procedure

Participants engaged in a one to one session in an outpatient clinic at the NCIMD where they were met by a study investigator. They were first administered the demographic questionnaire, followed by the semi‐structured interview. The interviews lasted between 34 and 56 minutes. Data were collected with the use of a digital voice recorder. Participants were advised in advance that all interviews would be audio recorded, and that the data would be transcribed and anonymized for the purposes of this study.

### 2.4 Data analysis

Audio recordings of semi‐structured interviews were initially transcribed verbatim and reviewed for accuracy. Thematic analysis, as outlined by Ziebland and McPherson,^18^ was then utilized to identify patterns in the dataset relevant to the aim of the study. Noteworthy phrases and paragraphs were highlighted and coded under different subthemes. These were then grouped together into larger, overarching key themes. Four key themes emerged (see Table 2).

**TABLE 2:** Themes and related quotes

Theme	Related quotes
Journey to diagnosis	Q1. *Participant 1*: “I was diagnosed 2 y ago, it was going on for 20 y” Q2. *Participant 1*. “For years you're told that you're mad like then all of a sudden someone says 'oh well there's actually a name on this” Q3. *Participant 4*: “The doctor dismissed it and I took on board that this was a mental problem” Q4. *Participant 5*: (*When paraphrasing their initial HCP*'*s comments*)“Yeah there's no such thing as that, a made up thing, you found it on Google” Q5. *Participant 1*: “…it almost feels like justification to me that like I'm not mad” Q6. *Participant 4*: “You still have a battle in your mind to stop the thoughts coming in that there is something wrong” Q7. *Participant 3*: “The staff have been great for me like, obviously it's very helpful” Q8. *Participant 1*: “I'm probably never going to get over this in a way” Q9. *Participant 4*: “Once you start talking about it you know the genie is out of the bottle and it enables you to be more talkative whereas for years in my case was from so many years bottled up where you didn't you couldn't talk about it”
Emotional impact	Q10. *Participant 1*: “I've always noticed peoples' breathing over the years…But it's something you're always a bit paranoid about where someone's breathing really heavy and you're thinking 'oh god why are they breathing so heavy…it's because of me” Q11. *Participant 4*: “I thought that when I go into the room or the church that because I had this fish smell, which I didn't realize what it was, that it would be noticeable that I was there and that might spoil the event” Q12. *Participant 2*: “I'd rather someone [call me an offensive name], but not for someone to say 'you smell'. That's like the worst thing, especially when you're clean…hyperclean. It rips you. I'd rather someone say anything else to me. It's an awful one. It's dirty….you feel dirty” Q13. *Participant 1*: (*In response to: “Any suicide attempts?”*) “No, no…thought about it, thought about it a good bit” Q14. *Participant 2*: “She's a lovely person but I found she didn't, which many people can't, like who can empathize completely to the disease” Q15. *Participant 4*: “The group is a really good thing…that people have been able to exorcise the demons”
Navigating daily life	Q16. *Participant 1*: “I would just try to avoid people, keep to myself and stuff like that” Q17. *Participant 2*: “Even getting the train up here, I booked my seat. I try and get the seat that's only two people sitting in and at the edge. Everything is premeditated” Q18. *Participant 3*: “I'm comfortable like I've said. I'm not going to let it get me down and I shouldn't” Q19. *Participant 2*: “For 2 or 3 y where I was just numb with depression is how I'd describe it….it consumed me, 99% of my life. It's all I ever thought about” Q20. *Participant 2*: You go about your daily life and nothing changes. It's still not known who's going to sit beside you, will they say something. And to be honest, most of the time people don't but you just…never know” Q21. *Participant 5*: “I didn't bother telling the lads. I'd be very careful about whom I tell which is probably stupid like because they probably all know…it's just such a personal thing” Q22. *Participant 4*: “The way I look at it, they can have their own thoughts on it whatever way it is. I'm telling them so they can understand my situation, or not understand it whichever”
Relationships and long‐term functioning	Q23. *Participant 1*: “I do have a large group of friends but I'm not really close to anyone” Q24. *Participant 5*: “Not that I don't want to but it's just kind of easier not to like I wouldn't trust people very easily anyway, so then to meet somebody or a group of new people. And to think that there are talking about you behind your back the same way that other people have and then, do you tell them, do you not tell them…just easier not to” Q25. *Participant 1*: “Yeah…yeah…it was a relief, it was almost like oh god at least I don't have worry about stress, get stressed out by these things” Q26. *Participant 2*: “Kind of like relief, even though after, there's realization that I've just ended a potential long term relationship with someone who I shouldn't have ended it with” Q27. *Participant 5*: “I've intentionally ruined things or caused a break up. Just the thoughts of being with someone or them being around me all the time was just too much for me, like growing up and over the last few years” Q28. *Participant 1*: “I would have had a few drinks in me so I would have used alcohol as a…just help…I suppose, to relieve the stress” Q29. *Participant 3*: “So that'd be the only thing that would hurt me the most would be if he gets it because I have it” Q30. *Participant 6*: “If we ever have children as like I think I could be a carrier and I like maybe if I ever do get pregnant, I don't know if he can get tested to see if he's now the chances are he's probably not a carrier, but that would be the only way that I'd wonder like I'd hate to pass this on to the child”

## 3 RESULTS

### 3.1 Journey to diagnosis

The journey from first noticing a malodor to eventual diagnosis of TMAU was a long, difficult one for patients, spanning a few years to several decades (see Table 2 for sample answers to questions presented). The participants' first incident with a malodor was a negative experience evoking varying feelings of dread, anxiety, fear, shame, and embarrassment (Table 2—Q1, Q2).

The majority of participants reported being dismissed by HCPs when they approached them about having a malodor. The participants were told there was nothing physically wrong with them or that it was in their head (Table 2—Q3, Q4).

When participants eventually managed to get a confirmed diagnosis of TMAU, all expressed a strong sense of relief and validation (Table 2—Q5).

While participants felt vindicated at having a diagnosis, many still felt that it did not change their day‐to‐day struggles with living with TMAU (Table 2—Q6).

Participants all reported positive experiences attending the services of the NCIMD. Participants noted a better understanding of their condition, boosted self‐esteem and confidence, and a sense of comfort knowing there was a support structure in place (Table 2—Q7).

While participants were appreciative of services provided to them, several remain dismayed at the prospect of living with a chronic condition with no cure, with one participant's outlook on the future remaining particularly bleak (Table 2—Q8).

Three out of six participants attend a TMAU support group run by the NCIMD team describing it as an important resource. Participants describe a shared sense of identity with other group members and feel able to share their own individual experiences of living with TMAU, while having these circumstances validated and appreciated (Table 2—Q9).

### 3.2 Emotional impact

For all participants, fear, anxiety, and paranoia are a daily struggle. Participants described being always acutely aware of their surroundings and who is near them. Unknown environments and strangers evoke strong feelings of fear and paranoia (Table 2—Q10).

This fear and paranoia has led participants to develop a dysfunctional type of thinking. Participants described numerous negative automatic thoughts such as catastrophizing, overgeneralizing, mind reading, and labeling. Common to several participants was a belief that their TMAU would intrude on others and brought up feelings of shame, guilt, and embarrassment (Table 2—Q11).

Many participants described the detrimental effect TMAU has on their self‐esteem, sense of self‐worth, and confidence in navigating daily life. Some participants describe receiving negative comments around malodor constantly, even after diagnosis, from strangers, family, friends, and work colleagues. Participants described how negative experiences with others left them with deep scars; to the extent that one participant reported suicidal ideation as a result of sustained negative comments in their youth (Table 2—Q12, Q13).

Several participants sought professional help within community mental health settings to address the emotional impact TMAU was having on their lives. They described mixed results, with many participants reporting an inability to empathize on the part of the mental health professional (Table 2—Q14).

Despite mixed experiences with mental health professionals, the three participants who attend the TMAU support group at the NCIMD reported positive experiences. They cite others' ability to empathize and understand TMAU, and similar past experiences as key positives of the group (Table 2—Q15).

### 3.3 Navigating daily life

Participants employ numerous coping mechanisms in order to navigate daily life with TMAU. All participants described their own ritualistic behaviors they engage in in order to ease their fears and anxiety. These included eating mints, excessive showering, and overusing perfume. Avoidance was described by almost all participants as a coping mechanism in group, enclosed, and social situations to varying degrees (Table 2—Q16).

Many participants reported organizing their lives around TMAU. This often involved meticulous strategic planning of their approach to situations and manipulation of the environment. These included designing workspace to minimize customer contact and sitting in specific areas on public transport (Table 2—Q17).

All participants described how TMAU is always on their mind. However, over half (n = 4) the group reported not being consumed by it in a way that negatively impacts on their everyday functioning (Table 2—Q18).

For the other participants, TMAU has consumed their lives and affects all aspects of daily living. One participant described how TMAU consumed their life in the past and how it continues to have an impact (Table 2—Q19, Q20).

Four out of six participants avoid discussing TMAU with others. This results in many of them often avoiding social situations as they do not want to face comments from others. One participant described how they carefully choose who to tell about their TMAU (Table 2—Q21). In contrast, another told many people about their TMAU diagnosis, believing it is up to others whether they choose to understand the disorder's impact or not (Table 2—Q22).

### 3.4 Relationship management and long‐term functioning

The impact of TMAU on relationships and long‐term functioning was another recurrent theme across participants. Almost all participants (n = 5) described TMAU as a source of stress in their relationships. One participant spoke of how they feel distanced from their peers, despite having a large group of friends (Table 2—Q23). Another remarked how it is easier to not get to know new people in the first instance (Table 2—Q24).

For several participants, avoidance was used to alleviate the stress associated with navigating relationships. One participant described the relief they felt when friends stopped inviting them to social events (Table 2—Q25). Several participants deliberately ended intimate relationships because of their TMAU, with two expressing a sense of relief at their ending (Table 2—Q26, Q27). While many participants use avoidance to manage relationship stress, one participant remarked that they rely on alcohol when interacting with others (Table 2—Q28).

Many participants spoke of how TMAU will influence their lives moving forward, with the potential impact of the disorder on their prospective children a particular concern. One participant remarked that the possibility of having passed TMAU to their child is their primary worry arising from their diagnosis (Table 2—Q29). Another participant stated that conservations with their partner around passing TMAU onto their future children were a burden on their mind (Table 2—Q30).

## 4 DISCUSSION

The aim of this study was to explore the lived experience of individuals with TMAU attending an adult metabolic center. Findings from this study support the argument that TMAU has a significant psychosocial impact on those with the condition.

### 4.1 Journey to diagnosis

This study found that individuals' early experience with malodor was a negative one with comments made about their malodor resulting in feelings of anxiety, shame, and embarrassment. This supports Chalmers et al^14^ who reported that children with TMAU attending school face embarrassment and ridicule resulting in low self‐esteem, social isolation, and exclusion. Mackay et al^4^ and Khan and Shagufta^12^ found similar results. Many of the participants in this study described a lack of awareness and understanding from their HCP of TMAU. Lateef and Marshall‐Lucette^19^ reported similar findings with almost all of their participants dismayed by the lack of awareness from HCPs. Christensen^20^ reported that due to HCPs not recognizing TMAU symptoms, diagnosis can take up to 10 years. This fits with the experience of participants in this study who were diagnosed between 4 years and several decades after reporting symptoms. It is important that HCPs are educated about this condition so that individuals are diagnosed and supported in a timely manner. Participants felt a great sense of relief and validation on receiving a diagnosis. Being able to make sense of their symptoms is helpful and learning that it is a medical condition and “not in my head” is important for living with it. Receiving support from the multidisciplinary team as well as being part of a TMAU support group is important to those in this study. It boosted their self‐esteem and confidence, and gave them better understanding of their condition. This may be one way to address the social isolation and social exclusion experienced by participants in this study and others.^14^, ^19^

### 4.2 Emotional impact

Much of the narratives explored in this study are consistent with previous findings. Ayesh et al^1^ reported that several of their participants described being ridiculed in school. Similar experiences were described by participants in this study. Unlike those in Ayesh et al,^1^ most participants in this study completed school and obtained a university degree. This suggests that although participants experienced difficulties in school with negative comments around malodor, the majority completed their education. Perhaps using coping strategies that in the moment may appear helpful but over time are dysfunctional (eg, avoiding spending time with peers, not socializing, sitting/standing away from others) was a way for individuals to manage this.

As participants left school and began working, they continued to receive negative comments from others. For many, it instilled fear, paranoia, anxiety, depression, shame, and embarrassment. It detrimentally impacted their experiences in forming interpersonal relationships, their ability to work and be able to follow their life goals and dreams. Similar detrimental effects have been reported by others.^11^, ^15^

Suicidal ideation and thoughts are an important risk factor to consider in TMAU. Consistent negative comments around one's odor can have a damaging impact on mental health. One participant in this study expressed suicidal thoughts and ideation when they were in school. Similar patterns have been observed in other studies lending support for the need for risk assessment and screening tools in this area.^1^, ^15^

While Ayesh et al^1^ noted that many people with TMAU and their families may benefit from individual counseling, that has not been the experience of participants in this study. They described a lack of understanding of TMAU from community mental health professionals. This would support the argument of a need for specialist counseling services for individuals with TMAU as suggested by Lateef and Marshall‐Lucette.^19^ Participants in this study placed a strong emphasis on the value of the TMAU support group run by the NCIMD, describing the boost in self‐esteem and confidence it gave them. This is something that should be offered at all IMD centers to support those living with this condition.

### 4.3 Navigating daily life

Respondents have reported ways of navigating through life using various coping strategies. These include obsessive rituals and strategic planning to minimalize feelings of fear and anxiety. This is reported to be common in this patient group.^12^, ^19^ Participants' experiences of negative comments from others around malodor lead to avoidance developing as a primary coping strategy. This resulted in social withdrawal and isolation, a rigid work/personal life routine, and detrimental impacts on relationships with others. The odor may not always be evident as patients may have learned to minimize the smell. It is important that healthcare providers be aware of this disorder. While TMAU is not life threatening, its severe impact on quality of life cannot be dismissed. Individuals struggle in their day‐to‐day functioning with constant feelings of fear, paranoia, dread, and consuming negative thoughts around malodor, demonstrating the detrimental psychological impact of the condition.^6^ Similar impairments reported by Lateef and Marshall‐Lucette^19^ support the strong need for specialized psychological intervention. The TMAU assessment tool tailored to assess treatment efficacy reported by Rutkowski et al,^16^ would help facilitate discussion between patients and clinicians around treatment plans. Many of the key themes found in this study are found in their tool, further supporting the need for patient‐centered care led by patients' experiences.

### 4.4 Relationship management and long‐term functioning

Previous research has reported TMAU patients as having difficulties in managing relationships.^1^, ^11^ This finding is consistent with experiences of participants in the current study, with almost all participants reporting that TMAU negatively influenced their relationships to varying degrees. Relationship difficulties spanned from work colleagues and peers, to family members and intimate partners. Several participants expressed a sense of relief at the avoidance of social interactions with friends, or at the deliberate ending of relationships in the case of intimate partners. Similar to the experiences of participants in the current study, negative consequences such as social isolation and reduced mental health have been observed in TMAU patients experiencing relationship difficulties.^1^, ^21^ This underlines the importance of psychological support for TMAU patients, with relationship functioning perhaps requiring particular attention.

In addition to navigating relationships, many participants were concerned about the impact TMAU would have on their lives moving forward. The possibility of passing the disorder down to their children was a particular burden on the mind of participants, a finding previously reported in TMAU patients.^19^

## 5 CONCLUSION

This research supports the urgent need for psychological support and intervention for people affected with TMAU post‐diagnosis. The journey to diagnosis is often long and difficult. Even after diagnosis, individuals struggle with living with TMAU. They may benefit from exploring their experiences with a psychologist who can support them. The psychologist may also work with the multidisciplinary team in providing patient‐centered care. The results suggest that counseling may not be sufficient to meet the needs of the individuals. Working with a clinical or health psychologist, or psychotherapist, may better meet their needs by being more goal‐specific (eg, psychoeducation about TMAU, behavior management, mindfulness based cognitive behavioral therapy, exploring identity and self‐esteem, as well as areas of shame, guilt, fear, anxiety, and depression). One of the limitations of this research is its small sample size. Of the six participants, three attend a hospital based support group for those with TMAU and are under the care of a consultant metabolic physician. The data therefore may not be generalizable. However, it is hoped that this study builds on the knowledge base of the lived experience of adults living with TMAU as there is very limited qualitative research in this area. Overall, this research aims to highlight the experience of adults with TMAU attending an Irish National Centre. Future research may explore the knowledge base of GPs and other HCPs regarding TMAU with a view to making recommendations in this area. Research could also examine the validity of psychology‐led support groups in coping with TMAU.

## CONFLICT OF INTEREST

The authors declare no conflict of interest.

## AUTHOR CONTRIBUTIONS

**Daniel Roddy**: Drafted and submitted ethics proposal, literature review, questionnaire design, data transcription, data collection and analysis, drafted article, critical review, article submission. **Philomena McCarthy**: Conceptualization of project, drafted and submitted ethics proposal, literature review, questionnaire design, data collection and analysis, contributed to article draft, critical review, article submission. **Darragh Nerney**: Data collection, transcription, and analysis, contributed to article draft, critical review. **Jennifer Mulligan‐Rabbitt**: Literature review, data transcription, data analysis, critical review. **Edwin Smith**: Biochemical data management and analysis, critical review. **Eileen P. Treacy**: Presented at ethics review board, drafted article, interpretation and analysis of data, critical review.

## ETHICS STATEMENT

Ethical approval was obtained from the Mater Misericordiae University Hospital Institutional Review Board. Ref: 1/378/2008.

## INFORMED CONSENT

All procedures followed were in accordance with the ethical standards of the responsible committee on human experimentation (institutional and national) and with the Helsinki Declaration of 1975, as revised in 2000. Informed consent was obtained from all patients for the study.
